# Advances in ovarian cancer treatment using a combination of statins with other drugs

**DOI:** 10.3389/fphar.2022.1048484

**Published:** 2023-01-04

**Authors:** Lei Xia, Shichao Ding, Xuezhen Wang, Xiaoyu Zhang, Lin Zhu, Hairong Zhang, Huirong Li

**Affiliations:** ^1^ Department of Pathology, Shandong University of Traditional Chinese Medicine, Jinan, China; ^2^ Department of Internal Medicine, The Third Affiliated Hospital of Shandong First Medical University, Jinan, China; ^3^ School of Chinese Medicine, Shandong University of Traditional Chinese Medicine, Jinan, China; ^4^ Department of Obstetrics and Gynecology, Shandong Provincial Third Hospital, Jinan, China

**Keywords:** statins, ovarian cancer, synergistic effect, HMGCoA reductase, progress

## Abstract

New anti-cancer drugs are constantly being developed, especially targeted drugs. Although these drugs have achieved significant clinical efficacy, they do not play a significant role in ovarian cancer. Moreover, the research cycle and costs of such drugs are often huge. The repositioning of conventional drugs has gradually become a concern. Statins, as traditional lipid-lowering drugs, play a role mainly by inhibiting HMGCR. In recent years, epidemiological studies and *in vitro* experiments have confirmed its anti-cancer effect, especially the effect of anti-ovarian cancer. The mutation rate of TP53 in ovarian cancer is as high as 95%, while HMGCR is often highly expressed in TP53 mutant tumors. However, the effect of prospective clinical trials is not ideal. This result seems understandable considering that it seems unrealistic for a lipid-lowering drug to completely inhibit tumor growth. Therefore, statins play more synergistic roles in the treatment of ovarian cancer. Because ovarian cancer is a highly heterogeneous tumor, it may be a good choice to deeply understand the mechanism of statins in the treatment of ovarian cancer and achieve precise treatment by combining it with other drugs.

## 1 Introduction

Ovarian cancer is a common malignant tumor reported in women and is also one of the gynecological tumors with the highest mortality rate. There are 239,000 new cases of ovarian cancer and 152,000 deaths of ovarian cancer every year in the world (GBD 2019 Stroke Collaborators, 2021). The incidence rate of Ovarian cancer in 2035 is estimated to be 371,000 (an increase of 55%), and the number of deaths is estimated to be 254,000 (an increase of 67%). Most ovarian cancers are diagnosed at stage Ⅲ and Ⅳ (Torre et al., 2018), when 5-year survival is less than 30% (Torre et al., 2018; Peres et al., 2019). Although routine treatment has demonstrated therapeutic outcomes, 70% of patients with ovarian cancer relapse and develop chemoresistance, and have a shorter survival time. New targeted drugs have been widely used in the treatment of tumors, but the effect is not ideal in the treatment of ovarian cancer, especially relapsed and drug-resistant ovarian cancer. It is therefore critical to identify novel drugs for patients who are dissatisfied with the clinical treatment effects. Due to the long development cycle and the high cost of new drugs, the repositioning of traditional drugs has gradually attracted people’s attention. Drug repositioning refers to the method of determining new target molecules and disease indications for approved drugs (National Academy of Sciences, 1975). When compared with traditional new drug research and development, it has a lower cost and shorter cycle.

Statins—hyperlipidemia drugs (Schachter, 2005)—have recently been discovered to have anti-cancer properties manifested through the inhibition of the cell cycle, anti-tumor proliferation (Kobayashi et al., 2015), induction of apoptosis and autophagy (Kobayashi et al., 2015; Hutchinson and Marignol, 2017), and increasing the chemotherapy sensitivity of tumors (Hutchinson and Marignol, 2017). Particularly, it plays an important role in anti-ovarian cancer. Some people believe that this is closely related to the characteristics of ovarian cancer and the target of statins. The main target of statins is HMGCR. The expression of HMGCR in TP53 mutant cells is generally increased, while the proportion of TP53 mutations in ovarian cancer cells is as high as 95%.

Several epidemiological studies and *in vitro* testing have also demonstrated that statins have anti-ovarian cancer effects (Liu et al., 2009; Xie et al., 2017; Li and ZHOU, 2018). For example, some studies (Xie et al., 2017; Urpilainen et al., 2018) have reported that the overall survival rate of patients taking statins is significantly higher than that of patients not taking statins. Even after the diagnosis of ovarian cancer, the overall survival rate of patients taking statins was significantly higher (Li and Zhou, 2018; Harding et al., 2019; Kim et al., 2022). However, some data showed the opposite results. For instance, some studies reported that statins can reduce the recurrence rate of ovarian cancer, but have no impact on the overall survival rate (Lavie et al., 2013; Bar et al., 2016; Chen et al., 2016). On the other hand, some studies believe that the use of statins can prevent the occurrence of ovarian cancer (Lavie et al., 2013; Zhang et al., 2019), while others believe that the use of statins cannot prevent the occurrence of ovarian cancer (Baandrup, 2015). Moreover, some studies found that the use of statins did not have a different effect on different ovarian cancer subtypes (Couttenier et al., 2017), but other studies showed that statins have a more obvious therapeutic effect on non-serous papillary epithelial cell ovarian cancer subtypes. It has been suggested that it may be related to the type of statin, lipophile and hydrophilic statins may have different effects on ovarian cancer (Jiao et al., 2020), and lipophile statins are more effective in reducing tumor progression because they have less liver selectivity than hydrophilic statins (Kato et al., 2010; Majidi et al., 2021).

In addition, the prospective clinical trials using statins to treat ovarian cancer have mostly been unsatisfactory (Altwairgi, 2015). The reason may be related to the type of ovarian cancer and the dosage of statins. On one hand, ovarian cancer exhibits high heterogeneity, the histological classification of ovarian cancer is complex with over 100 types having been identified (Granström et al., 2008). Ovarian cancer treatment pathways are targeted at the type and depend on histopathological diagnosis. Ovarian cancer can be divided into epithelial, sex cord-stromal, and germ-cell tumor types. Among these, epithelial ovarian cancer is the most common one and can be further categorized into serous, mucinous, endometrioid, and clear cell cancers according to their cell types. In addition, ovarian cancer can be low-grade or high-grade, based on the degree of malignancy. The biological characteristics of the two types of tumors are extremely different as are their genetic backgrounds. The common mutations in HGSOC were TP53 (96%), BRCA1/2 (23%) and HRD (homologous recombination defect) mutation (50%); The common mutations in clear cell carcinoma are PIK3CA, ARD1A, PTEN and microsatellite instability (MSI); Common mutations in endometrioid carcinoma are CTNNB1, ARID1A, PTEN, MSI; The common mutations in mucinous carcinoma are KRAS, HER2 and CDKN2A mutations; In LGSOC, mutations such as BRAF, KRAS, NRAS, ERBB2 and PIK3CA are more common (Schachter, 2005).

The anti-tumor effect of statins is not limited to HMGCR inhibition, but it has different anti-tumor effects against different tumors. On the other hand, in several studies, the drug dosage is often utilized for hypercholesterolemia treatment, which may have resulted in the plasma concentration of the drug being lower than that necessary for inducing cell apoptosis *in vitro* tests (Robinson et al., 2013). However, large-dose statins may cause myalgia and other adverse effects.

It is undeniable that, although some studies have shown that there is no difference in the survival time between the simple use of statins and the simple use of chemotherapy drugs, epidemiological investigations often combine statins on the basis of conventional treatment. Therefore, the anti-tumor effect of statins is mainly reflected by the combination with other therapies, or it mainly plays an auxiliary role. Therefore, it is important to better understand its anti-ovarian cancer mechanism and the synergistic role with other drugs, which has a significant role in improving the overall clinical efficacy and reducing adverse drug reactions.

This study examined research on the mechanism of statin treatment of ovarian cancer and how the therapeutic effects can be improved through drug combinations. This study aimed to obtain ideas for application in future fundamental research and clinical treatment (Table 1).

**TABLE 1:** Synergistic effect of statins with other drugs in the treatment of ovarian cancer.

	Drugs	Cell models	Potential	References
			The mechanism	
1	Zoledronic acid + fluvastatin	22 pre-treated ovarian carcinomas	Activates the tricarboxylic acid cycle, autophagy	Robinson et al. (2013)
2	Fluvastatin + cisplatin	CAOV3 SKOV3	Induction of apoptosis	Mctaggart (2006)
			Inhibition of GGT	
			Inhibition of Ras, Rho, Rab	
3	Lovastatin + doxorubicin	A2780ADR	Inhibiting P-glycoprotein.	De Wolf et al. (2017)
4	Oxysterols + statins	SKOV-3	Inhibition of SREBPs	Lipper et al. (2019)
		OVCAR-8		
5	Carboplatin + simvastatin	A2780, Ovcar-5, Ovcar-8 Igrov-1	Unclear	Liu et al. (2009)
6	Paclitaxel + simvastatin	A2780, Ovcar-5, Ovcar-8 Igrov-1	Unclear	Liu et al. (2009)
7	Paclitaxel + simvastatin	ES2	Inhibition of VDCA1 binding to tubulin	Abdullah et al. (2019), Kobayashi et al. (2022)
8	Panobinostat + simvastatin	ES2	Inhibition of HDAC	Kobayashi et al. (2022)
9	Prednisolone + pitavastatin	Ovsaho, Cov-318, Cov-362, Ovcar-3, Ovcar-4	Inhibition of MVA pathway;	Svensmark and Brakebusch (2019)
			HMGCR and FDPS were reduced	
10	ABT-737 + pitavastatin	Igrov-1	PARP cleavage	Jeon and Osborne (2012)
			Bcl-xL was reduced	
11	Pictilisib + pitavastatin	OVCAR3	Inhibiting NF-κB	Jeon and Osborne (2012)
			PTEN modulation	
12	Atorvastatin + JQ1	Hey, SKOV3	Inhibiting c-Myc	Jones et al. (2017)

## 2 Treatment by inhibiting the mevalonic acid (MVA) pathway

**FIGURE 1:**
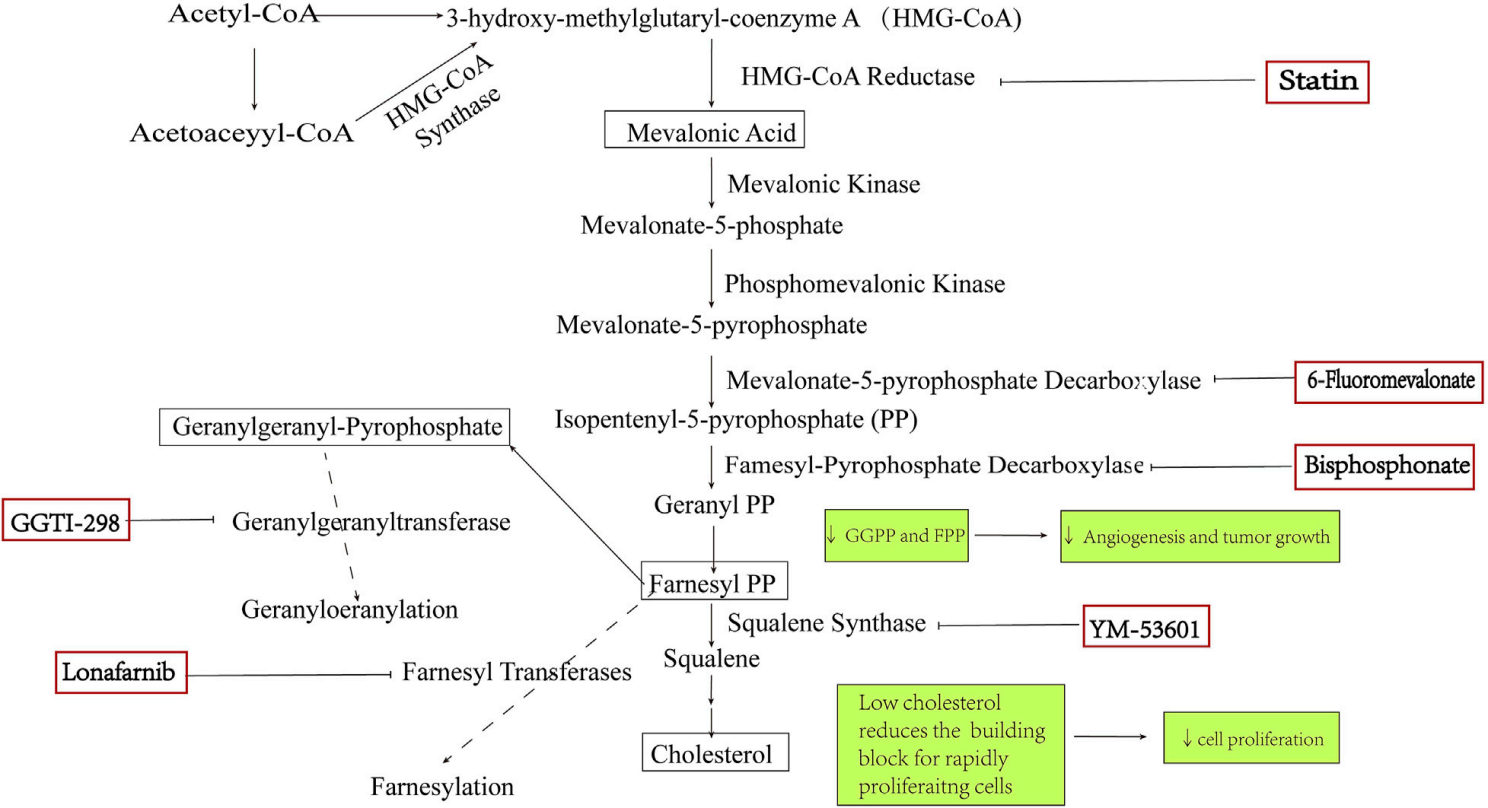
MVA pathway and the related inhibitors.

Statins’ anti-tumor effect mainly blocks the MVA pathway by inhibiting HMGCR. HMGCR is considered a metabolic oncogene that promotes tumor growth and development (Clendening et al., 2010). HMGCR is commonly seen in TP53-mutated tumor cells, and the TP53 mutation rate in ovarian cancer cells is as high as 95% (Freed-Pastor and Prives, 2016). De Wolf et al. investigated 12 ovarian tumor cell lines and found that HMGCR was upregulated in ovarian tumor cells when compared to that in normal ovarian surface epithelial cells (De Wolf et al., 2017). Combining HMGCR with other MVA pathway antagonists may achieve a sound synergistic anti-ovarian cancer effect (Figure 1).

### 2.1 Synergistic blocking of protein isoprenylation by statins and MVA pathway inhibitor

FPP, GGPP, and cholesterol are intermediate and end-products of the MVA pathway. FPP and GGPP, for example, can provide isopentenyl groups, thereby allowing isoprenylation of multiple small GTPase binding proteins and anchoring them to the cell membrane (e.g., Ras and Rho). Membrane localization is, therefore, necessary for these proteins to function, and several oncogenes interfering with the isoprenylation of these proteins can have an anti-tumor effect (Mctaggart, 2006; Svensmark and Brakebusch, 2019).

Ibandronate and zoledronic acid are FPP synthase inhibitors, which limit the production of FPP and GGPP and thus inhibit the isoprenylation of the relevant proteins (Kobayashi et al., 2015; Pletcher et al., 2017). These drugs can inhibit various tumors (Green, 2004), and the combination of statins with zoledronic acid has synergistic inhibitory effects on the growth of ovarian cancer cells (Abdullah et al., 2017).

Other targets in the isoprenylation pathway have also been investigated to identify their effects on ovarian cancer. The cumulative results demonstrated that lonafarnib (farnesyl transferase inhibitor) and GGTI-298 (geranylgeranyl transferase inhibitor) can inhibit the proliferation of SKOV3 and OVCAR5 cells by inhibiting isoprenylation. By inhibiting the MVA pathway, 6-fluoromevalonate (mevalonate pyrophosphate decarboxylasyrophose inhibitor) and YM-53601 (squalene synthase inhibitor) could inhibit tumor cells. However, a higher dosage of 6-fluoromevalonate and YM-53601 is warranted (Kobayashi et al., 2017). By inhibiting the MVA pathway, drugs can induce cell autophagy. Nevertheless, whether these drugs and statins have a synergistic anti-ovarian cancer effect needs further verification (Figure 1).

### 2.2 Combination of statins and cisplatin blocks the small GTPase signaling pathway

**FIGURE 2:**
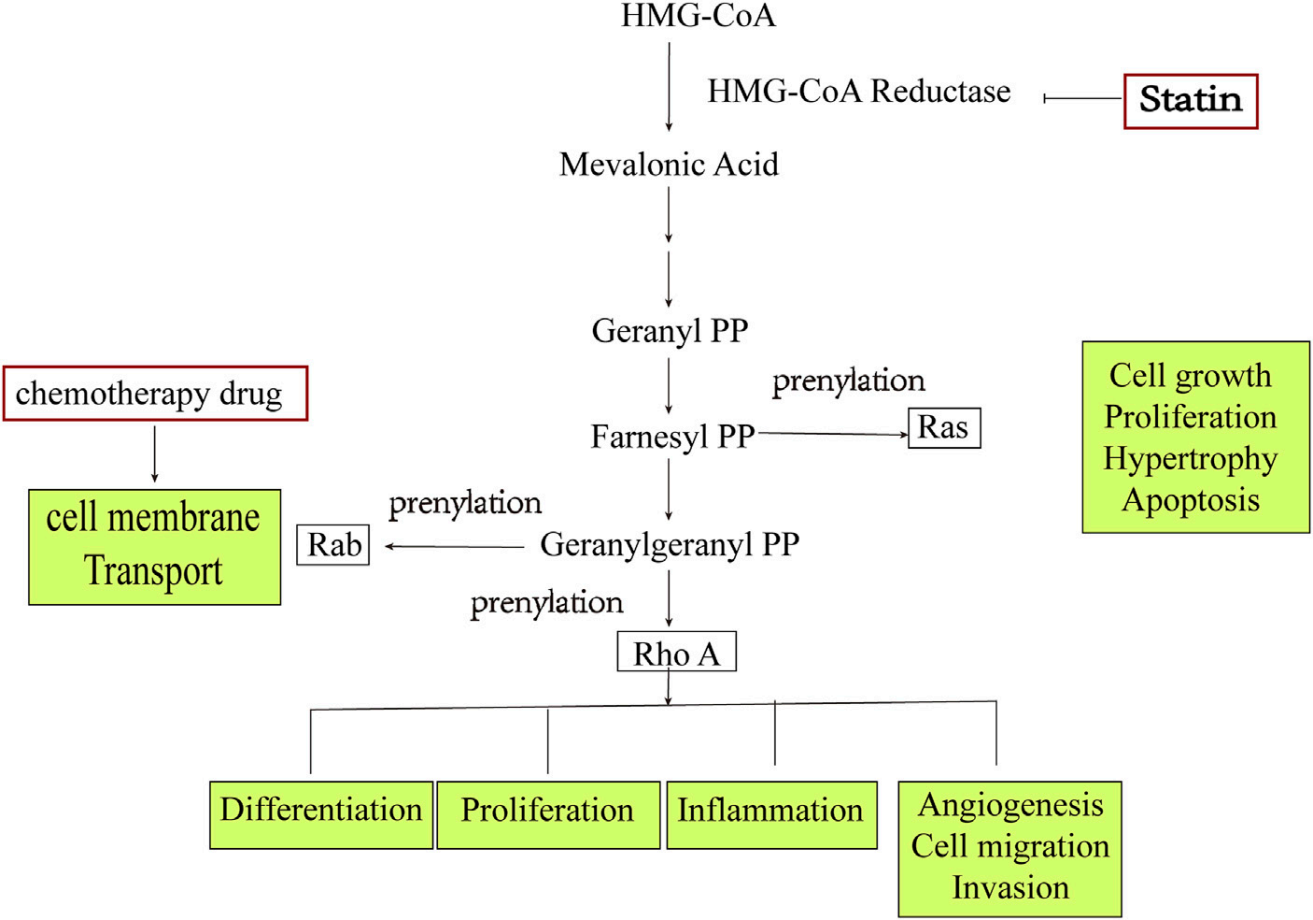
Effect of statins on small GTPase and its potential antitumor effects.

Among the diverse isoprenylated proteins, the small GTPase superfamily, which comprises Ras, Rho, and Rab, has attracted significant attention. In addition, it has been demonstrated that lovastatin and simvastatin can inhibit the proliferation of ovarian cancer cells, induce apoptosis, and cause cell-cycle arrest (Kato et al., 2010; Martirosyan et al., 2010), which is closely related to the inhibition of Ras, Rho, and Rab (Taylor-Harding et al., 2010). Meanwhile, it was found that fluvastatin and cisplatin possess synergistic cytotoxicity (Taylor-Harding et al., 2010). Accordingly, a hypothesis was proposed that Rab1 protein regulated the cell membrane transport and cell growth and that the detection of unmodified Rab1 protein might lead to the synergistic effect of fluvastatin and cisplatin combination therapy. Robinson et al. (2013) reported that statins alone could reduce cell activity and proliferation and increase cell apoptosis and autophagy. A superposition effect was discovered when the experimental cells were exposed to statins and carboplatin or paclitaxel, although the specific mechanism was not explored in depth. Nevertheless, some scholars (Martirosyan et al., 2010) claim that the combination of lovastatin and cisplatin has synergistic effects only at high concentrations (Figure 2).

### 2.3 Combination of statins and prednisone promotes apoptosis of ovarian cancer cells

Previous studies (Abdullah et al., 2019) have demonstrated that prednisolone has minimal influence on the caspase 3/7 activity in ovarian cancer cells. However, pivastatin combined with prednisolone significantly increased the caspase activation when compared to that by pivastatin alone. Scholars have also investigated the expression of MVA pathway-related genes as prednisolone regulates gene expression by binding to glucocorticoid receptors. They found that neither pitavastatin nor prednisolone alone had any effect on HMGCR, GGTⅠ-β, IDI1, MVD, and FDPS levels, but they could reduce the GGTⅡ-β expression. Pitavastatin with prednisolone reduced the levels of GGTⅡ-β and resulted in significantly lower levels of HMGCR and FDPS. In addition, the combination of prednisolone and pivastatin led to a significant PARP accumulation relative to that of either drug individually (Abdullah et al., 2019).

## 3 Inhibition of tumor growth by metabolic reprogramming

**FIGURE 3:**
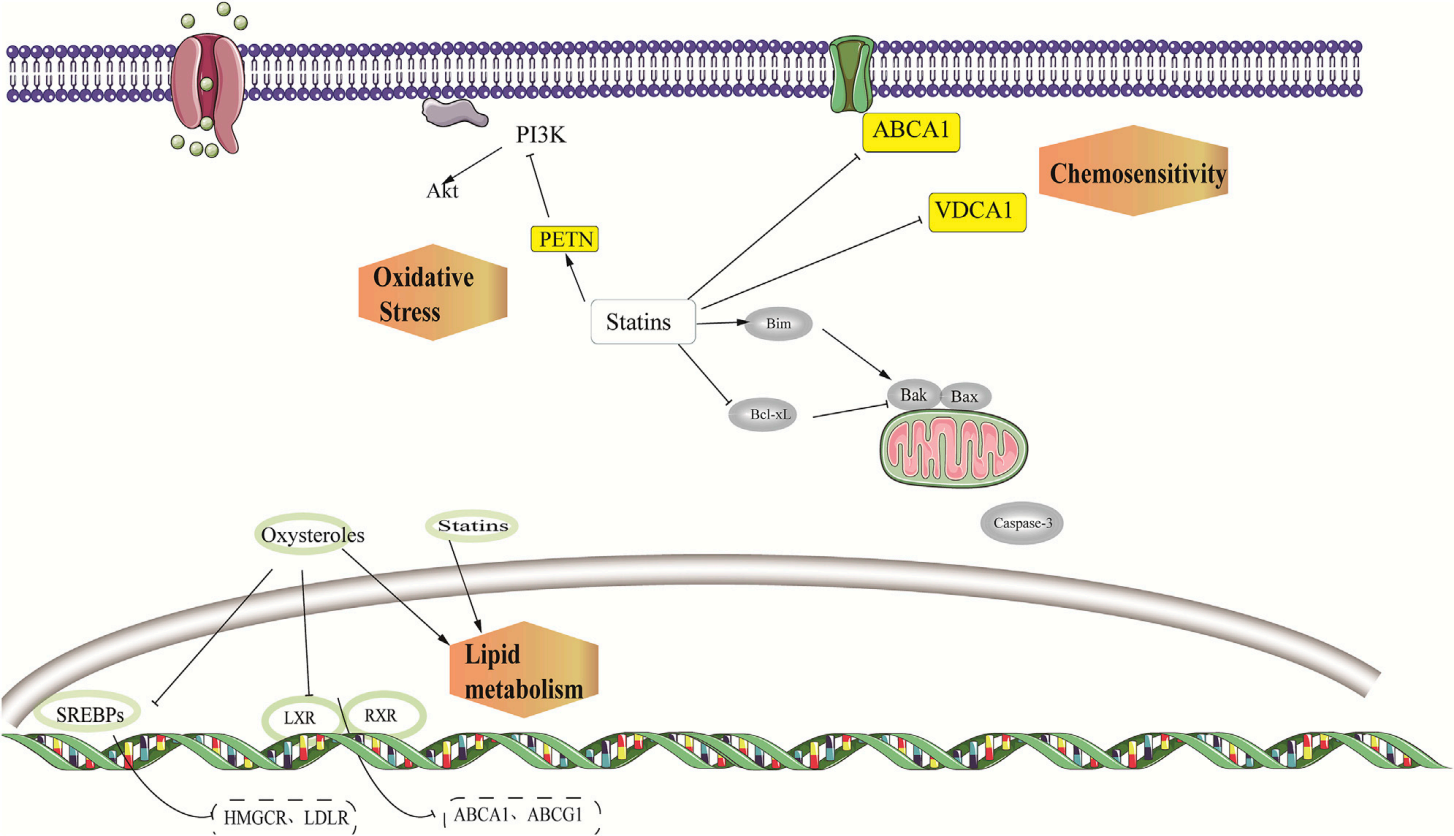
Other potential antitumor effects of statins.

Aerobic glycolysis is common in tumor cells. Aerobic glycolysis can lead to increased acidic substances, inhibiting immune cell function, and promoting tumor metastasis. Acetyl CoA increased, whereas lactate decreased significantly in statins-treated cells. It was also found through the analysis of other metabolites that metabolites related to the tricarboxylic acid cycle increased significantly and that their metabolic characteristics were similar to those of normal cells (Figure 3).

### 3.1 Inhibition of tumor growth by VDAC1

*VDAC1* was demonstrated to be a positively correlated gene with statin response in ovarian cancer cells (Kobayashi et al., 2022). VDAC1 encodes a 30-kDa channel protein found in the outer mitochondrial membrane (Mannella and Bonner, 1975). VDAC may transport substrates for energy metabolism from the cytoplasm to the intermembrane zone and metabolites from the intermembrane zone to the cytoplasm as it can non-selectively penetrate substances with a molecular weight of about 6,000 Da. Therefore, it is an essential protein for effective energy metabolism in the mitochondria (Rostovtseva et al., 2006). By binding to hexokinase, VDAC regulates glycolysis and interacts with mitochondrial respiration (the rate-limiting enzyme of glycolysis) (Pastorino et al., 2002; Mazure, 2017). VDAC1 expression is upregulated in several human cancer cell lines when compared to normal cell lines. VDAC1 is thus a potential therapeutic target for cancer. In addition, statins have a regulating effect on VDAC1 (Baandrup, 2015; Lipper et al., 2019).

Paclitaxel and panopistat have synergistic anti-ovarian cancer effects (Kobayashi et al., 2022). Paclitaxel is an effective anti-tumor drug that can stoichiometrically and specifically bind to the β-tubulin subunit in tubulin. The VDAC 1 expression may be involved in the synergistic effect of statins and paclitaxel. Tubulin can regulate VDAC *via* a functional interaction between dimer tubulin and VDAC1 (Rostovtseva et al., 2008). Tubulin and VDAC interact at the C-terminal tail of tubulin, which then penetrates the lumen of the VDAC barrel and functions as a plug. Because of the length of the C-terminal tail, which is about the length of the VDAC pore, the C-terminal tail is negatively charged and the VDAC-related domain is positively charged, which contributes to tubulin and VDAC binding (Mazure, 2017). Statins are one of the promising drugs for targeting VDAC; hence, the synergistic antitumor effect of paclitaxel and statins may be related to microtubules and VDAC binding (Reina And De Pinto, 2017; Fang And Maldonado, 2018).

### 3.2 Cholesterol metabolism affected the proliferation of ovarian cancer cells

Elevated cholesterol levels could decrease progression-free survival in ovarian cancer patients (Li et al., 2010). Statins reduce cholesterol synthesis by inhibiting the MVA pathway. Statins can induce HMGCR increase through the sterol reaction in some statin-resistant tumor cells, rather than in statin-sensitive cells. This type of feedback depends on SREBPs (Jeon and Osborne, 2012). Oxysterol inhibits SREBP activation by preventing protein processing and nuclear transport. Indeed, statins-oxysterol combination treatment could significantly enhance statin cytotoxicity in ovarian cancer cells (Casella et al., 2014). This drug combination is effective against statins-sensitive cells and has a synergistic effect on statin-resistant cells. The mechanism may be related to the inhibition of SREBPs activity, and, thus, a decrease in the intracellular cholesterol content. LXRs bind to oxysterol and active LXRs can inhibit the growth of ovarian cancer cells. Notably, 25HC was used in this study, and the results showed that 25HC had no significant effect on promoting apoptosis or proliferation in ovarian cancer cells (Casella et al., 2014), despite past research suggesting that HC might promote tumor growth. 27HC was tested in another study (He et al., 2019), which could be metabolized by cholesterol through CYP27A1. Moreover, 27HC has been shown to inhibit the growth of ovarian cancer cells. Whether 27HC can cooperate with the anti-tumor effect of statins warrants further verification. Moreover, 27HC may promote the metastasis of ovarian cancer cells, which needs further consideration.

## 4 Other mechanisms

### 4.1 Induction of cell apoptosis by the PI3K/AKT signal pathway

The PI3K/AKT signaling pathway promotes tumor cell proliferation and anti-apoptosis and is overexpressed in 45% of high-grade ovarian cancer (Cancer Genome Atlas Research Network, 2011). Statins can inhibit PI3K activation by inhibiting NF-κB, which promotes the expression of PTEN and reduces AKT activation (Ghosh-Choudhury et al., 2010; Miraglia et al., 2012). The investigation of OVCAR3, OVCAR8, A2780, and Igrov-1 constitutively activated by the PI3K/AKT signaling pathway revealed that the combination of pictilisib and pivastatin had a synergistic inhibitory effect on OVCAR3 but an antagonistic effect on A2780 and Igrov (De Wolf et al., 2018). This observation may be attributed to the low expression of PTEN in A2780 and Igrov cells, which results in an inability to reduce AKT activation.

Simvastatin inhibited the PI3K/AKT pathway in SKOV3 and HEY cells, increased the active oxygen level to cause DNA damage, induced ER stress, and reduced the VEGF expression, thus playing an anti-proliferation and metastasis role in ovarian cancer (Stine et al., 2016). Moreover, after lovastatin intervention, total glutathione, reduced glutathione, and oxidized glutathione levels in the ovarian cancer cells were significantly decreased (Kobayashi et al., 2017) (Figure 3).

### 4.2 Induction of cell apoptosis by the Bcl-2 superfamily

The Bcl-2 family includes pro-apoptotic proteins, anti-apoptotic proteins, and pro-apoptotic proteins of BH-3-only. Herein, the anti-apoptotic protein acted by binding with the pro-apoptotic proteins or BH-3-only proteins. Past studies (De Wolf et al., 2018) have demonstrated that pivastatin had no significant effect on Bcl-2 alone, although it could promote the expression of the pro-apoptotic protein Bim and decrease the expression of the anti-apoptotic protein Bcl-XL. Moreover, Bcl-XL was highly expressed in ovarian cancer (Lee et al., 2019).

ABT-737 is a BH3 mimetic inhibitor, and the combination of pivastatin and ABT-737 significantly promoted ovarian cancer cell death (De Wolf et al., 2018). Pivastatin may increase Bim and decrease Bcl-xL, accelerating ABT-737, antagonizing Bcl-xL, and inducing Bim release, which finally activated the cell-specific apoptosis process. A relatively significant synergistic effect can be found in Igrov cells, but none in A2780 cells, which may be due to the low expression of Bcl-xL but a high expression of Mcl-1 in A2780 cells. Furthermore, ABT-737 could inhibit Bcl-xL, Bcl-2, and Bcl-w, but not Mcl-1, Bcl-B, or Bfl-1 (Witham et al., 2007) (Figure 3). However, some studies have determined that simvastatin could inhibit TNF-α-induced NF-κB activation, leading to the downregulation of cyclin D1, Bcl-2, MMP9, and VEGF (Ahn et al., 2007).

### 4.3 Anti-tumor chemoresistance mediated by p-gp

ABCB1 encodes p-gp, and increasing its expression can accelerate drug excretion, which is one of the critical mechanisms of tumor chemoresistance (Waghray and Zhang, 2018). Past studies (Martirosyan et al., 2010) noted no significant synergistic effect between statins and cisplatin or doxorubicin and statins for chemotherapy-sensitive A2780 cells; however, there was a significant synergistic effect between lovastatin and adriamycin in chemo-resistant A2780ADR cells. Moreover, adriamycin treatment increased the expression of p-gp in A2780 cells, while statins could affect the inhibitory effect of the p-gp overexpression. Meanwhile, the adriamycin level of the chemo-resistant cell strain rose. However, because the p-gp expression was not considered in the cells, the combination of statins and adriamycin demonstrated no significant synergistic effect in treating A2780 cells. Therefore, the authors proposed that statins had therapeutic effects on ovarian cancer *via* the MVA pathway, could antagonize drug chemoresistance by inhibiting p-glycoprotein and had synergistic effects with the use of chemotherapeutic drugs (Figure 3).

### 4.4 Anti-tumor effect mediated by Myc

C-Myc is associated with both cell proliferation and the cell cycle. Statins may induce cell growth arrest by inhibiting c-Myc. In some studies, three statins were used to intervene in ovarian cancer cells OVCAR8 and its multidrug chemo-resistant cell strain NCI-ADR/RES. The results demonstrated that all three statins could inhibit cell proliferation without causing cell apoptosis while also arresting the G1 and S phases. Meanwhile, statins accelerated c-Myc degradation and inhibited c-Myc protein synthesis, indicating that statins interfere with c-Myc biosynthesis, which exists not only in chemotherapy-sensitive ovarian cancer cells but also in multi-chemo-resistant cells. JQ1 is a c-Myc inhibitor, and Jones et al. found that JQ1 combined with Atorvastatin had a synergistic inhibitory effect on c-Myc and a synergistic anti-ovarian cancer effect (Jones et al., 2017).

Hence, we believe that statins can be clinically applied in chemo-resistant tumor cells. However, after ceasing the administration, the cell proliferation gradually recovered, indicating the significance of statin action time in cancer treatment (Rao and Rao, 2021) (Figure 3).

## 5 Discussion

Although several studies have demonstrated that statins play an important role in the prevention and treatment of ovarian cancer, most of these studies are retrospective in nature and often combined with the use of statins on the basis of conventional treatment (Table 2). The results of ovarian cancer treated with statins alone are full of contradictions (Robinson et al., 2013; Chen et al., 2016; Wang et al., 2019), and some studies even believe that statins can promote the occurrence of ovarian cancer (Desai et al., 2018). On the one hand, this contradiction is related to the positioning of statins. It is difficult to imagine that an anti-lipid drug can completely kill tumor cells. Therefore, statins may need to be applied together with other drugs to better play an anti-tumor role. On the other hand, it may also be related to the high heterogeneity of ovarian cancer.

**TABLE 2:** Summary of the evidence regarding impact of statin therapy on risk and survival of ovarian cancer.

Type of study	Size of population	Primary outcome variable	Conclusion	References
Retrospective cohort	8,629	Risk of ovarian cancer	Improved survival among use statin after diagnosis, especially in endometrioid cancer and those who use statin for long.	Feng et al. (2021)
Case control study	Cases, 4,103; matched controls, 58,706	Risk of epithelial ovarian cancer	Decreased risk seen in mucinous ovarian cancer. No association with epithelial subtype	Baandrup et al. (2015)
Retrospective cohort	442	Progression-free survival and disease-specific survival	Improved survival among statin users was not seen except in non-serous papillary epithelial ovarian cancer	Habis et al. (2014)
Case control study	Cases, 12; matched controls, 126	Risk of ovarian cancer and survival	Decreased risk along with improved survival was reported	Lavie et al. (2013)
Retrospective cohort	73,336	Risk of ovarian cancer	Non-significant decrease in ovarian cancer risk was found	Yu et al. (2009)
Retrospective cohort	126	Progression-free survival and overall survival	Improved survival was seen in statin users	Elmore et al. (2008)
Retrospective cohort	361,859	Risk of ovarian cancer	No association was found	Friedman et al. (2008)
Case control study	Cases, 91; controls, 7,393	Risk of ovarian cancer	Statins have no substantial effect on ovarian cancer risk	Abdullah et al. (2019)
Retrospective cohort	997	Risk of ovarian cancer	No difference in frequency of cancer between statin users/non-users was reported	Clearfield et al. (2001)
Retrospective cohort	421	Mortality rate of ovarian cancer	Pre-diagnostic use of statins was observed to be associated with decreased mortality	Urpilainen et al. (2018)

Some studies (Baandrup et al., 2015; Verdoodt et al., 2017; Feng et al., 2021) believe that statins have obvious effects in the treatment of endometrioid and mucinous carcinoma, but not in serous or mucinous carcinoma. In serous ovarian cancer, statins have a protective effect only after diagnosis (Hanley et al., 2021). Therefore, the protective effect of statins may be limited to certain specific subtypes, but, even for a certain subtype, the biological characteristics of cancer cells are different.

At present, the sample size of the ongoing or completed clinical trials is small. Although the sample size of the retrospective trials can be relatively large, the data collected is not precise enough. Therefore, the data collection of relevant retrospective studies should be more detailed and targeted. For example, not only the type of statin, the type of ovarian cancer, but also the conventional treatment method should be considered, and relevant clinical studies should look for appropriate molecular markers on the basis of existing studies, rather than simply relying on tissue type.

## 6 Conclusion and prospect

The anti-tumor effect of statins not only affects the MVA pathway by inhibiting HMGCR, but also may affect cell proliferation, apoptosis and drug resistance through metabolic reprogramming, Bcl-2 family and other pathways. Moreover, statins can exert synergistic anti-ovarian cancer effect by combining with a variety of drugs. However, the current clinical studies mainly focus on the combined use of statins and chemotherapy drugs. Combination with other drugs is less common. A thorough understanding of the working mechanism of statins is expected to facilitate the achievement of “precision treatment” by using them either alone or in combination, thereby improving the overall clinical efficacy. Furthermore, suitable tumor markers are essential and should be investigated in the future.

## Abbreviations

HMG-CoA, 3-hydroxy-3-methyglutaryl-coenzyme A; HMGCR, HMG-CoA reductase; MVA, mevalonic acid; PARP, poly ADP-ribose polymerase; FPP, farnesyl pyrophosphate; GGPP, geranylgeranyl pyrophosphate; GGT, geranylgeranyltransferase; ABCB1, ATP-binding cassette subfamily B member 1; MVD, mevalonate (diphospho) decarboxylase; VDCA1, voltage-dependent anion-selective channel protein 1; FDPS, Farnesyl diphosphate synthase; IDI, isopentenyl-diphosphate delta Isomerase; SREBPs, sterol regulatory element-binding proteins.

## References

[B1] Abdullah M. I. Abed M. N. Khanim F. Richardson A. (2019). Screening a library of approved drugs reveals that prednisolone synergizes with pitavastatin to induce ovarian cancer cell death. Sci. Rep. 9 (1), 9632. 10.1038/s41598-019-46102-1 31270377 PMC6610640

[B2] Abdullah M. I. Abed M. N. Richardson A. (2017). Inhibition of the mevalonate pathway augments the activity of pitavastatin against ovarian cancer cells. Sci. Rep. 7 (1), 8090. 10.1038/s41598-017-08649-9 28808351 PMC5556066

[B3] Ahn K. S. Sethi G. Aggarwal B. B. (2007). Simvastatin potentiates TNF-alpha-induced apoptosis through the down-regulation of NF-kappaB-dependent antiapoptotic gene products: Role of IkappaBalpha kinase and TGF-beta-activated kinase-1. J. Immunol. Baltim. Md 178 (4), 2507–2516. 10.4049/jimmunol.178.4.2507 17277159

[B4] Altwairgi A. K. (2015). Statins are potential anticancerous agents (review) [J]. Oncol. Rep. 33 (3), 1019–1039. 10.3892/or.2015.3741 25607255

[B5] Baandrup L. Dehlendorff C. Friis S. Olsen J. H. Kjær S. K. (2015). Statin use and risk for ovarian cancer: A Danish nationwide case-control study. Br. J. cancer 112 (1), 157–161. 10.1038/bjc.2014.574 25393364 PMC4453615

[B6] Baandrup L. (2015). Drugs with potential chemopreventive properties in relation to epithelial ovarian cancer-a nationwide case-control study. Dan. Med. J. 62, B5117. 26183052

[B7] Bar D. Lavie O. Stein N. Feferkorn I. Shai A. (2016). The effect of metabolic comorbidities and commonly used drugs on the prognosis of patients with ovarian cancer. Eur. J. obstetrics, Gynecol. reproductive Biol. 207, 227–231. 10.1016/j.ejogrb.2016.09.005 27890326

[B8] Cancer Genome Atlas Research Network (2011). Integrated genomic analyses of ovarian carcinoma. Nature 474 (7353), 609–615. 10.1038/nature10166 21720365 PMC3163504

[B9] Casella C. Miller D. H. Lynch K. Brodsky A. S. (2014). Oxysterols synergize with statins by inhibiting SREBP-2 in ovarian cancer cells. Gynecol. Oncol. 135 (2), 333–341. 10.1016/j.ygyno.2014.08.015 25134999 PMC5516475

[B10] Chen H. Y. Wang Q. Xu Q. H. Yan L. Gao X. F. Lu Y. H. (2016). Statin as a combined therapy for advanced-stage ovarian cancer: A propensity score matched analysis. BioMed Res. Int. 2016, 9125238. 10.1155/2016/9125238 27975064 PMC5128698

[B11] Clearfield M. Downs J. R. Weis S. Whitney E. J. Kruyer W. Shapiro D. R. (2001). Air force/Texas coronary atherosclerosis prevention study (AFCAPS/TexCAPS): Efficacy and tolerability of long-term treatment with lovastatin in women. J. women's health & gender-based Med. 10 (10), 971–981. 10.1089/152460901317193549 11788107

[B12] Clendening J. W. Pandyra A. Boutros P. C. El Ghamrasni S. Khosravi F. Trentin G. A. (2010). Dysregulation of the mevalonate pathway promotes transformation. Proc. Natl. Acad. Sci. U. S. A. 107 (34), 15051–15056. 10.1073/pnas.0910258107 20696928 PMC2930553

[B13] Couttenier A. Lacroix O. Vaes E. Cardwell C. R. De Schutter H. Robert A. (2017). Statin use is associated with improved survival in ovarian cancer: A retrospective population-based study. PloS one 12 (12), e0189233. 10.1371/journal.pone.0189233 29261726 PMC5736195

[B14] De Wolf E. Abdullah M. I. Jones S. M. Menezes K. Moss D. M. Drijfhout F. P. (2017). Dietary geranylgeraniol can limit the activity of pitavastatin as a potential treatment for drug-resistant ovarian cancer. Sci. Rep. 7 (1), 5410. 10.1038/s41598-017-05595-4 28710496 PMC5511264

[B15] De Wolf E. De Wolf C. Richardson A. (2018). ABT-737 and pictilisib synergistically enhance pitavastatin-induced apoptosis in ovarian cancer cells. Oncol. Lett. 15 (2), 1979–1984. 10.3892/ol.2017.7516 29434898 PMC5778268

[B16] Desai P. Wallace R. Anderson M. L. Howard B. V. Ray R. M. Wu C. (2018). An analysis of the association between statin use and risk of endometrial and ovarian cancers in the Women's Health Initiative. Gynecol. Oncol. 148 (3), 540–546. 10.1016/j.ygyno.2018.01.006 29422345 PMC5896309

[B17] Elmore R. G. Ioffe Y. Scoles D. R. Karlan B. Y. (2008). Impact of statin therapy on survival in epithelial ovarian cancer. Gynecol. Oncol. 111 (1), 102–105. 10.1016/j.ygyno.2008.06.007 20698078

[B18] Fang D. Maldonado E. N. (2018). VDAC regulation: A mitochondrial target to stop cell proliferation. Adv. cancer Res. 138, 41–69. 10.1016/bs.acr.2018.02.002 29551129 PMC6234976

[B19] Feng J. L. Dixon-Suen S. C. Jordan S. J. Webb P. M. (2021). Statin use and survival among women with ovarian cancer: An Australian national data-linkage study. Br. J. cancer 125 (5), 766–771. 10.1038/s41416-021-01460-4 34135470 PMC8405606

[B20] Freed-Pastor W. Prives C. (2016). Targeting mutant p53 through the mevalonate pathway. Nat. Cell Biol. 18 (11), 1122–1124. 10.1038/ncb3435 27784901

[B21] Friedman G. D. Flick E. D. Udaltsova N. Chan J. Quesenberry C. P. Jr Habel L. A. (2008). Screening statins for possible carcinogenic risk: Up to 9 years of follow-up of 361, 859 recipients. Pharmacoepidemiol. drug Saf. 17 (1), 27–36. 10.1002/pds.1507 17944002

[B22] GBD 2019 Stroke Collaborators (2021). Global, regional, and national burden of stroke and its risk factors, 1990-2019: A systematic analysis for the global burden of disease study 2019 [J]. Lancet Neurology 20 (10), 795–820. 10.1016/S1474-4422(21)00252-0 34487721 PMC8443449

[B23] Ghosh-Choudhury N. Mandal C. C. Ghosh-Choudhury N. Ghosh Choudhury G. (2010). Simvastatin induces derepression of PTEN expression via NFkappaB to inhibit breast cancer cell growth. Cell. Signal. 22 (5), 749–758. 10.1016/j.cellsig.2009.12.010 20060890 PMC2826504

[B24] Granström C. Sundquist J. Hemminki K. (2008). Population attributable fractions for ovarian cancer in Swedish women by morphological type. Br. J. cancer 98 (1), 199–205. 10.1038/sj.bjc.6604135 18071361 PMC2359681

[B25] Green J. R. (2004). Bisphosphonates: Preclinical review. Oncol. 9, 3–13. 10.1634/theoncologist.9-90004-3 15459425

[B26] Habis M. Wroblewski K. Bradaric M. Ismail N. Yamada S. D. Litchfield L. (2014). Statin therapy is associated with improved survival in patients with non-serous-papillary epithelial ovarian cancer: A retrospective cohort analysis. PloS one 9 (8), e104521. 10.1371/journal.pone.0104521 25118694 PMC4131884

[B27] Hanley G. E. Kaur P. Berchuck A. Chase A. Grout B. Deurloo C. M. (2021). Cardiovascular medications and survival in people with ovarian cancer: A population-based cohort study from British columbia, Canada. Gynecol. Oncol. 162 (2), 461–468. 10.1016/j.ygyno.2021.05.021 34090707 PMC9398205

[B28] Harding B. N. Delaney J. A. Urban R. R. Weiss N. S. (2019). Use of statin medications following diagnosis in relation to survival among women with ovarian cancer [J]. Cancer epidemiology, biomarkers & prevention : A publication of the American association for cancer research. cosponsored by Am. Soc. Prev. Oncol. 28 (7), 1127–1133. 10.1158/1055-9965.EPI-18-1194 31064757

[B29] He S. Ma L. Baek A. E. Vardanyan A. Vembar V. Chen J. J. (2019). Host CYP27A1 expression is essential for ovarian cancer progression. Endocrine-related cancer 26 (7), 659–675. 10.1530/ERC-18-0572 31048561 PMC6824983

[B30] Hutchinson J. Marignol L. (2017). Clinical potential of statins in prostate cancer radiation therapy. Anticancer Res. 37 (10), 5363–5372. 10.21873/anticanres.11962 28982844

[B31] Jeon T. I. Osborne T. F. (2012). SREBPs: Metabolic integrators in physiology and metabolism. Trends Endocrinol. metabolism TEM 23 (2), 65–72. 10.1016/j.tem.2011.10.004 22154484 PMC3273665

[B32] Jiao X. F. Li H. L. Jiao X. Y. Guo Y. C. Zhang C. Yang C. S. (2020). Ovary and uterus related adverse events associated with statin use: An analysis of the FDA adverse event reporting system. Sci. Rep. 10 (1), 11955. 10.1038/s41598-020-68906-2 32686733 PMC7371681

[B33] Jones H. M. Fang Z. Sun W. Clark L. H. Stine J. E. Tran A. Q. (2017). Atorvastatin exhibits anti-tumorigenic and anti-metastatic effects in ovarian cancer *in vitro* . Am. J. cancer Res. 7 (12), 2478–2490. 29312801 PMC5752688

[B34] Kato S. Smalley S. Sadarangani A. Chen-Lin K. Oliva B. Branes J. (2010). Lipophilic but not hydrophilic statins selectively induce cell death in gynaecological cancers expressing high levels of HMGCoA reductase. J. Cell. Mol. Med. 14 (5), 1180–1193. 10.1111/j.1582-4934.2009.00771.x 19432822 PMC3822754

[B35] Kim D. S. Ahn H. S. Kim H. J. (2022). Statin use and incidence and mortality of breast and gynecology cancer: A cohort study using the national health insurance claims database. Int. J. cancer 150 (7), 1156–1165. 10.1002/ijc.33869 34751444

[B36] Kobayashi Y. Kashima H. Rahmanto Y. S. Banno K. Yu Y. Matoba Y. (2017). Drug repositioning of mevalonate pathway inhibitors as antitumor agents for ovarian cancer. Oncotarget 8 (42), 72147–72156. 10.18632/oncotarget.20046 29069775 PMC5641118

[B37] Kobayashi Y. Kashima H. Wu R. C. Jung J. G. Kuan J. C. Gu J. (2015). Mevalonate pathway antagonist suppresses formation of serous tubal intraepithelial carcinoma and ovarian carcinoma in mouse models. Clin. cancer Res. official J. Am. Assoc. Cancer Res. 21 (20), 4652–4662. 10.1158/1078-0432.CCR-14-3368 PMC4609247 26109099

[B38] Kobayashi Y. Takeda T. Kunitomi H. Chiwaki F. Komatsu M. Nagai S. (2022). Response predictive markers and synergistic agents for drug repositioning of statins in ovarian cancer. Pharm. (Basel, Switz. 15 (2), 124. 10.3390/ph15020124 PMC8880614 35215239

[B39] Lavie O. Pinchev M. Rennert H. S. Segev Y. Rennert G. (2013). The effect of statins on risk and survival of gynecological malignancies. Gynecol. Oncol. 130 (3), 615–619. 10.1016/j.ygyno.2013.05.025 23718932

[B40] Lee J. M. Minasian L. Kohn E. C. (2019). New strategies in ovarian cancer treatment. Cancer 125, 4623–4629. 10.1002/cncr.32544 31967682 PMC7437367

[B41] Li A. J. Elmore R. G. Chen I. Y. Karlan B. Y. (2010). Serum low-density lipoprotein levels correlate with survival in advanced stage epithelial ovarian cancers. Gynecol. Oncol. 116 (1), 78–81. 10.1016/j.ygyno.2009.09.027 19822357 PMC4437689

[B42] Li X. Zhou J. (2018). Impact of postdiagnostic statin use on ovarian cancer mortality: A systematic review and meta-analysis of observational studies. Br. J. Clin. Pharmacol. 84 (6), 1109–1120. 10.1111/bcp.13559 29453799 PMC5980397

[B43] Lipper C. H. Stofleth J. T. Bai F. Sohn Y. S. Roy S. Mittler R. (2019). Redox-dependent gating of VDAC by mitoNEET. Proc. Natl. Acad. Sci. U. S. A. 116 (40), 19924–19929. 10.1073/pnas.1908271116 31527235 PMC6778226

[B44] Liu H. Liang S. L. Kumar S. Weyman C. M. Liu W. Zhou A. (2009). Statins induce apoptosis in ovarian cancer cells through activation of JNK and enhancement of Bim expression. Cancer Chemother. Pharmacol. 63 (6), 997–1005. 10.1007/s00280-008-0830-7 18766339

[B45] Majidi A. Na R. Jordan S. J. De Fazio A. Webb P. M. OPAL Study Group (2021). Statin use and survival following a diagnosis of ovarian cancer: A prospective observational study. Int. J. cancer 148 (7), 1608–1615. 10.1002/ijc.33333 33034053

[B46] Mannella C. A. Bonner W. D. J. R. (1975). Biochemical characteristics of the outer membranes of plant mitochondria. Biochimica biophysica acta 413 (2), 213–225. 10.1016/0005-2736(75)90105-4 172151

[B47] Martirosyan A. Clendening J. W. Goard C. A. Penn L. Z. (2010). Lovastatin induces apoptosis of ovarian cancer cells and synergizes with doxorubicin: Potential therapeutic relevance. BMC cancer 10, 103. 10.1186/1471-2407-10-103 20298590 PMC2847546

[B48] Mazure N. M. (2017). VDAC in cancer. Biochimica biophysica acta Bioenergetics 1858 (8), 665–673. 10.1016/j.bbabio.2017.03.002 28283400

[B49] Mctaggart S. J. (2006). Isoprenylated proteins, Isoprenylated proteins [J]. Cell. Mol. life Sci. CMLS 63 (3), 255–267. 10.1007/s00018-005-5298-6 16378247 PMC11136304

[B50] Miraglia E. HöGBERG J. Stenius U. (2012). Statins exhibit anticancer effects through modifications of the pAkt signaling pathway. Int. J. Oncol. 40 (3), 867–875. 10.3892/ijo.2011.1223 21994073

[B51] National Academy of Sciences (1975). “The national academies collection: Reports funded by national institutes of health [M],” in Drug repurposing and repositioning: Workshop summary Washington (DC): National Academies Press. 2014, Copyright by the National Academy of Sciences. All rights reserved. 2014.

[B52] Pastorino J. G. Shulga N. Hoek J. B. (2002). Mitochondrial binding of hexokinase II inhibits Bax-induced cytochrome c release and apoptosis. J. Biol. Chem. 277 (9), 7610–7618. 10.1074/jbc.M109950200 11751859

[B53] Peres L. C. Cushing-Haugen K. L. KöBEL M. Harris H. R. Berchuck A. Rossing M. A. (2019). Invasive epithelial ovarian cancer survival by histotype and disease stage. J. Natl. Cancer Inst. 111 (1), 60–68. 10.1093/jnci/djy071 29718305 PMC6335112

[B54] Pletcher M. J. Pignone M. Jarmul J. A. Moran A. E. Vittinghoff E. Newman T. (2017). Population impact & efficiency of benefit-targeted versus risk-targeted statin prescribing for primary prevention of cardiovascular disease. J. Am. Heart Assoc. 6 (2), e004316. 10.1161/JAHA.116.004316 28188251 PMC5523747

[B55] Rao P. S. Rao U. S. (2021). Statins decrease the expression of c-Myc protein in cancer cell lines. Mol. Cell. Biochem. 476 (2), 743–755. 10.1007/s11010-020-03940-2 33070276

[B56] Reina S. De Pinto V. (2017). Anti-cancer compounds targeted to VDAC: Potential and perspectives. Curr. Med. Chem. 24 (40), 4447–4469. 10.2174/0929867324666170530074039 28554318

[B57] Robinson E. Nandi M. Wilkinson L. L. Arrowsmith D. M. Curtis A. D. M. Richardson A. (2013). Preclinical evaluation of statins as a treatment for ovarian cancer. Gynecol. Oncol. 129 (2), 417–424. 10.1016/j.ygyno.2013.02.003 23402903

[B58] Rostovtseva T. K. Kazemi N. Weinrich M. Bezrukov S. M. (2006). Voltage gating of VDAC is regulated by nonlamellar lipids of mitochondrial membranes. J. Biol. Chem. 281 (49), 37496–37506. 10.1074/jbc.M602548200 16990283

[B59] Rostovtseva T. K. Sheldon K. L. Hassanzadeh E. Monge C. Saks V. Bezrukov S. M. (2008). Tubulin binding blocks mitochondrial voltage-dependent anion channel and regulates respiration. Proc. Natl. Acad. Sci. U. S. A. 105 (48), 18746–18751. 10.1073/pnas.0806303105 19033201 PMC2596221

[B60] Schachter M. (2005). Chemical, pharmacokinetic and pharmacodynamic properties of statins: An update. Fundam. Clin. Pharmacol. 19 (1), 117–125. 10.1111/j.1472-8206.2004.00299.x 15660968

[B61] Stine J. E. Guo H. Sheng X. Han X. Schointuch M. N. Gilliam T. P. (2016). The HMG-CoA reductase inhibitor, simvastatin, exhibits anti-metastatic and anti-tumorigenic effects in ovarian cancer. Oncotarget 7 (1), 946–960. 10.18632/oncotarget.5834 26503475 PMC4808044

[B62] Svensmark J. H. Brakebusch C. (2019). Rho GTPases in cancer: Friend or foe? [J]. Oncogene 38 (50), 7447–7456. 10.1038/s41388-019-0963-7 31427738

[B63] Taylor-Harding B. Orsulic S. Karlan B. Y. Li A. J. (2010). Fluvastatin and cisplatin demonstrate synergistic cytotoxicity in epithelial ovarian cancer cells. Gynecol. Oncol. 119 (3), 549–556. 10.1016/j.ygyno.2010.08.017 20837358

[B64] Torre L. A. Trabert B. Desantis C. E. Miller K. D. Samimi G. Runowicz C. D. (2018). Ovarian cancer statistics, 2018 [J]. CA a cancer J. Clin. 68 (4), 284–296. 10.3322/caac.21456 PMC6621554 29809280

[B65] Urpilainen E. Marttila M. Hautakoski A. Arffman M. Sund R. Ilanne-Parikka P. (2018). Prognosis of ovarian cancer in women with type 2 diabetes using metformin and other forms of antidiabetic medication or statins: A retrospective cohort study. BMC cancer 18 (1), 767. 10.1186/s12885-018-4676-z 30055585 PMC6064082

[B66] Verdoodt F. Kjaer Hansen M. Kjaer S. K. Pottegard A. Friis S. Dehlendorff C. (2017). Statin use and mortality among ovarian cancer patients: A population-based cohort study. Int. J. cancer 141 (2), 279–286. 10.1002/ijc.30738 28411390

[B67] Waghray D. Zhang Q. (2018). Inhibit or evade multidrug resistance P-glycoprotein in cancer treatment. J. Med. Chem. 61 (12), 5108–5121. 10.1021/acs.jmedchem.7b01457 29251920 PMC6281405

[B68] Wang Y. Ren F. Song Z. Chen P. Liu S. Ouyang L. (2019). Statin use and the risk of ovarian and endometrial cancers: A meta-analysis. BMC cancer 19 (1), 730. 10.1186/s12885-019-5954-0 31340777 PMC6657066

[B69] Witham J. Valenti M. R. De-Haven-Brandon A. K. Vidot S. Eccles S. A. Kaye S. B. (2007). The Bcl-2/Bcl-XL family inhibitor ABT-737 sensitizes ovarian cancer cells to carboplatin. Clin. cancer Res. official J. Am. Assoc. Cancer Res. 13 (23), 7191–7198. 10.1158/1078-0432.CCR-07-0362 18056200

[B70] Xie W. Ning L. Huang Y. Liu Y. Zhang W. Hu Y. (2017). Statin use and survival outcomes in endocrine-related gynecologic cancers: A systematic review and meta-analysis. Oncotarget 8 (25), 41508–41517. 10.18632/oncotarget.17242 28489569 PMC5522329

[B71] Yu O. Boudreau D. M. Buist D. S. Miglioretti D. L. (2009). Statin use and female reproductive organ cancer risk in a large population-based setting. Cancer causes control CCC 20 (5), 609–616. 10.1007/s10552-008-9271-1 19043788 PMC3041638

[B72] Zhang Q. S. Deater M. Phan N. Marcogliese A. Major A. Guinan E. C. (2019). Combination therapy with atorvastatin and celecoxib delays tumor formation in a Fanconi anemia mouse model. Pediatr. blood cancer 66 (1), e27460. 10.1002/pbc.27460 30255556 PMC6249055

